# Flux Balance Analysis of Mycolic Acid Pathway: Targets for Anti-Tubercular Drugs

**DOI:** 10.1371/journal.pcbi.0010046

**Published:** 2005-10-14

**Authors:** Karthik Raman, Preethi Rajagopalan, Nagasuma Chandra

**Affiliations:** Bioinformatics Centre, Supercomputer Education and Research Centre, Indian Institute of Science, Bangalore, India; Boston University, United States of America

## Abstract

*Mycobacterium tuberculosis* is the focus of several investigations for design of newer drugs, as tuberculosis remains a major epidemic despite the availability of several drugs and a vaccine. Mycobacteria owe many of their unique qualities to mycolic acids, which are known to be important for their growth, survival, and pathogenicity. Mycolic acid biosynthesis has therefore been the focus of a number of biochemical and genetic studies. It also turns out to be the pathway inhibited by front-line anti-tubercular drugs such as isoniazid and ethionamide. Recent years have seen the emergence of systems-based methodologies that can be used to study microbial metabolism. Here, we seek to apply insights from flux balance analyses of the mycolic acid pathway (MAP) for the identification of anti-tubercular drug targets. We present a comprehensive model of mycolic acid synthesis in the pathogen *M. tuberculosis* involving 197 metabolites participating in 219 reactions catalysed by 28 proteins. Flux balance analysis (FBA) has been performed on the MAP model, which has provided insights into the metabolic capabilities of the pathway. In silico systematic gene deletions and inhibition of InhA by isoniazid, studied here, provide clues about proteins essential for the pathway and hence lead to a rational identification of possible drug targets. Feasibility studies using sequence analysis of the *M. tuberculosis* H37Rv and human proteomes indicate that, apart from the known InhA, potential targets for anti-tubercular drug design are AccD3, Fas, FabH, Pks13, DesA1/2, and DesA3. Proteins identified as essential by FBA correlate well with those previously identified experimentally through transposon site hybridisation mutagenesis. This study demonstrates the application of FBA for rational identification of potential anti-tubercular drug targets, which can indeed be a general strategy in drug design. The targets, chosen based on the critical points in the pathway, form a ready shortlist for experimental testing.

## Synopsis

*M. tuberculosis,* a deadly human pathogen, owes many of its unique qualities to its thick, waxy coat, containing fatty acids called mycolic acids. Several front-line drugs used for treating tuberculosis indeed inhibit mycolic acid synthesis. Understanding the biochemical pathway that makes these compounds is therefore of great interest. Availability of the genome sequence and various computational methods enable us to study pathways as whole functional units, rather than having to infer from the study of individual proteins. Here, we present a comprehensive identification of the components of the mycolic acid pathway and represent it mathematically based on reaction stoichiometry. Such models are amenable to perturbations and simulations using flux balance analysis, allowing the study of pathways from a metabolic capacity perspective, and yielding information about reaction fluxes. The perturbations studied here are in silico gene knock-outs and drug effects, which led us to identify genes essential to the pathway and hence for survival of the pathogen. The results are in good agreement with essentiality determined through experimental genetics. Such essential genes can be good targets for drug design, especially when they do not have homologues in the human proteome. FBA followed by sequence analyses have resulted in identification of potential anti-tubercular drug targets.

## Introduction

Genomics is rapidly changing the very foundation of several aspects of drug discovery research, one of them being a systems biology and bioinformatics approach for rational identification of drug targets. It is now possible to carry out a metabolic analysis of a system to gain insights into fundamental molecular mechanisms of several processes such as those that are critical for the survival of the pathogen. Here, we seek to apply such an approach to identify targets for designing anti-tubercular drugs. Despite the availability of several drugs and the Bacillus Calmette-Guérin vaccine, tuberculosis remains a major health concern worldwide, warranting identification of new drug targets for the design of more efficacious drugs.

The mycobacterial cell wall is distinctive and is associated with the pathogenicity of *Mycobacterium tuberculosis* [1–4]. The three polymers in the cell wall, arabinogalactan-mycolate [5] covalently linked with peptidoglycan and trehalose dimycolate, provide a thick layer that protects the tubercle bacillus from general antibiotics and the host's immune system [6]. The synthesis of mycolic acids—which are long-chain α-alkyl-β-hydroxy fatty acids, the major constituents of this protective layer—has been shown to be critical for the survival of *M. tuberculosis* [7]. InhA (EC 1.3.1.9, enoyl-[acyl-carrier-protein] reductase), involved in mycolic acid synthesis, also turns out to be the target for front-line anti-tubercular drugs [8], such as isoniazid [9] and ethionamide [10].

The mycolic acid pathway (MAP) has been of great interest, and a large amount of biochemical and genetic information is available in the literature, in addition to the entire genome sequence of *M. tuberculosis*. It is possible to exploit these large volumes of data to construct an in silico model of the pathway, which can then be simulated and analysed [11]. Constructing such models forms an important step in understanding the underlying molecular mechanisms of disease, and facilitates rational approaches to drug design. Several computational methods have emerged in recent years to simulate biochemical models, which aid in the systems approach to understanding pathways, processes, and whole-cell metabolism [12–18]. Flux balance analysis (FBA), a stoichiometric analysis technique, has been applied to study the metabolic capabilities of several systems [19,20], which has provided useful insights into cellular behaviour, including response to perturbations such as gene deletions [21,22].

Given the biological importance of mycolic acids, it would be useful to understand the behaviour of the pathway as a whole and of its individual components, both in a normal mycobacterial cell as well as upon perturbations, such as when a drug is acting upon it. We have therefore built a model of the MAP, represented it as a stoichiometric matrix, and performed FBA of this model, to gain insights into the critical steps of the pathway. Further, we have used the knowledge gained from these analyses for rational identification of putative drug targets and estimated their appropriateness by sequence analysis.

## Results/Discussion

### Model Description

**Figure 1:**
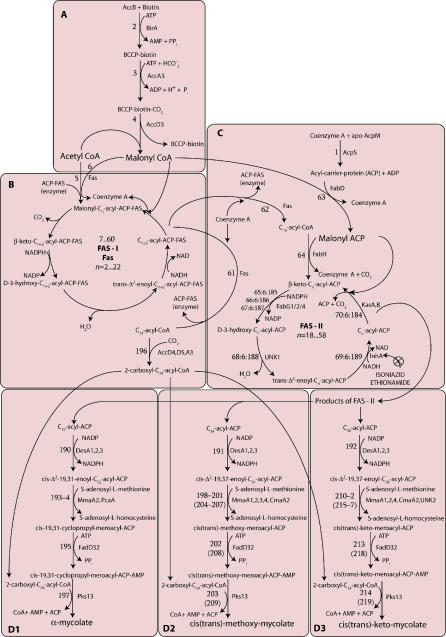
Schematic Diagram of the MAP in *M. tuberculosis* (A–D) refer to the four sub-pathways (see text). The key metabolites are indicated in larger type. Proteins catalysing each reaction are indicated to the right of the reaction arrows, while the reaction numbers are indicated to the left. Reaction cycles have been indicated as, for instance, 65:6:185 (for *fabG1*), which is to be interpreted as 65, 71, 77, ..., 185. ⊗ indicates inhibition of InhA by isoniazid and ethionamide in the pathway. The reaction numbers in parentheses indicate reactions for the *trans* forms in (D2) and (D3).

The model of the MAP built here (Figure 1), containing 219 reactions and 197 metabolites, mediated through 28 proteins (Table 1), is according to our knowledge more complete and accurate with annotations from the latest literature than that from any other publicly available resource. The model in Systems Biology Markup Language (SBML) format is available as Dataset S1. The list of reactions has also been given in a flat file as Dataset S2. The biosynthesis of mycolic acids can be considered as made up of four sub-pathways: (A) production of malonyl CoA, (B) fatty acid synthase-I (FAS-I) pathway, (C) fatty acid synthase-II (FAS-II) pathway, and (D) condensation of FAS-II and FAS-I products into α- (D1), methoxy- (D2), and keto-mycolic acids (D3). FAS-I and FAS-II (B and C sub-pathways) are dependent upon the production of malonyl CoA (produced in A). The products of B and C are then converted into different mycolic acids in D.

**Table 1:**
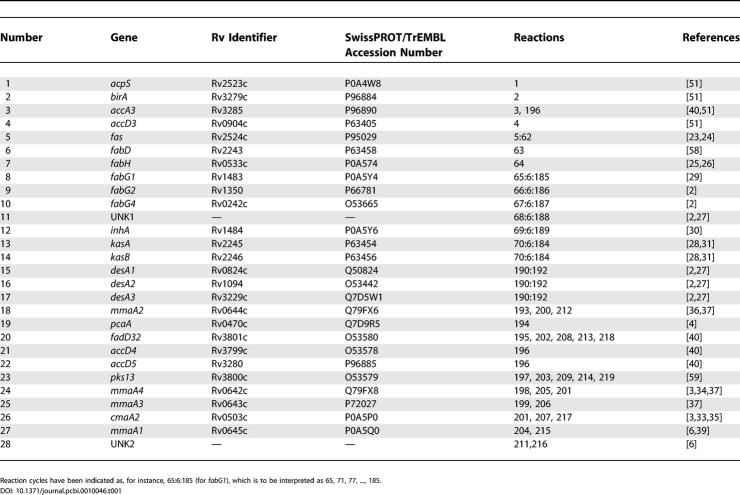
Table of Proteins and Their Corresponding Genes in MAP and Sources for Inference of Their Reactions

**Figure 2:**
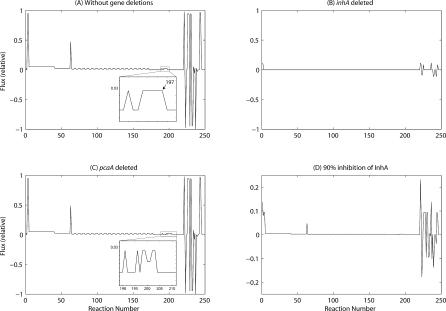
Flux Distributions Obtained from FBA Using the MAP Model and Objective Function **c**_1_ (A) in an unperturbed state, (B) upon deletion of *inhA*, (C) upon deletion of *pcaA,* and (D) upon inhibition of InhA. Insets in (A) and (C) refer to enlarged versions of the indicated portions. Note that the scale for (D) is different. It may be noted that the lines joining the various flux points have been drawn to aid in discerning the flux peaks clearly; the lines as such have no physical significance.

The FAS-I system, present predominantly in eukaryotes, is capable of de novo fatty acid synthesis, whereas the FAS-II system in mycobacteria, although similar to that in other bacteria, elongates the products of FAS-I, resulting in the production of meromycolates, key precursors of mycolic acids [2]. The basic reactions in FAS-I and FAS-II are a repetition of a cycle of four reactions, each cycle culminating in the extension of the alkyl chain by a two-carbon unit (Figure 2). The FAS-I enzyme is a single polypeptide with multiple domains catalysing a cycle of reactions to generate short-chain acyl CoA esters [23]. FAS-I exhibits a bimodal product distribution: C_16_ to C_18_ and C_24_ to C_26_ acyl CoAs [24]. These form the substrates for the FAS-II reaction cycle and the polyketide synthase enzyme, respectively. β-ketoacyl-synthase III forms a pivotal link between FAS-I and FAS-II [25,26]. The FAS-II system is composed of four enzyme reactions iteratively converting C_16_-acyl CoA to C_58_-acyl-ACP (meromycolate) [27–31].

Except for reactions 190–192, experimental data clearly indicating the involvement of the appropriate proteins are available in literature. However, clear identification of the proteins referred to as UNK1 and UNK2 is not yet available. Although no explicit experimental evidence is available for reactions 190–192, the involvement of *desA1, desA2,* and *desA3* has been suggested based on sequence annotations [27] and indirect experimental evidence [32], thus justifying their inclusion in this model. The *cis*-unsaturated meromycolate chain further undergoes cyclo-propanation, processing for keto- and methoxy-mycolic acids [3,4,33–39] and Claisen condensation with the FAS-I product C_24_-acyl CoA [6,40], to yield α-, methoxy-, or keto-mycolic acids, as shown in Figure 1D.

Biochemical characterisation of mycolic acids in *M. tuberculosis* H37Rv cell cultures clearly indicate that α-mycolate is the predominant mycolic acid and it comprises as much as 49% of the mycolates in the cell wall, whereas methoxy- and keto-mycolates are present in smaller quantities of 27% and 24%, respectively [41]. These data have been considered during FBA of the MAP model.

### FBA

The MAP system outlined here, though involving only 28 proteins, has 197 metabolites participating in 219 reactions with high interconnectivity, and thus benefits from a systematic FBA. The system considered here, though relatively small in comparison to genome-scale metabolic models previously studied by FBA [19–22], is still complete in its own right (analogous to a separate module) and gives profound insights into mycolic acid metabolism. More importantly, with a single pathway in question, the objective functions for optimisation can be better defined and have specific biological relevance that can be related to experimental data quite readily.

The stoichiometric matrix for this system is of size 197 × 247. The vector **v** (for details, see Materials and Methods) has 247 fluxes, including 28 exchange fluxes:


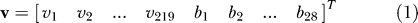


Objective function: Mycolic acids are known to play a key role in the structural integrity of the mycobacterial cell wall and have been shown to be critical both for growth [42–44] of the bacillus and its pathogenicity [1–4] by several experiments. These data imply that the mycobacterial cell would be geared toward optimal production of mycolic acids, in terms of maximal molar yield of the appropriate mycolic acids for a given genotype.

Given that the cell wall contains different types of mycolates in varying proportions, the optimal production of mycolates can be captured in two different ways: (a) only the most important mycolate is produced, and (b) the known ratios of different mycolates are fixed. We encode these two scenarios as two objective functions, **c**_1_ and **c**_2_, respectively:


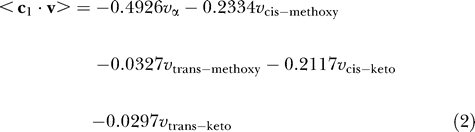


and


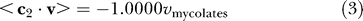


where *v*_mycolates_ represents the flux of a hypothetical reaction:

0.4926 α-mycolate + 0.2334 *cis*-methoxy-mycolate

+ 0.0327 *trans*-methoxy-mycolate + 0.2117 *cis*-keto-mycolate

+ 0.0297 *trans*-keto-mycolate → mycolate-biomass.

The coefficients of the fluxes of α-mycolates, *cis*-methoxy and *trans*-methoxy mycolates, as well as *cis*-keto and *trans*-keto mycolates indicated in the above equations are based on the biochemical data about α-, methoxy-, and keto-mycolates present in the cell wall in the ratio of 1.0:0.54:0.49, with the *cis* forms dominating the *trans* forms of the methoxy- and keto-mycolates, in a ratio of 1:0.14 [41]. Production of different mycolates follows much of the same pathway but differs only in the end stages (Figure 1D), suggesting that they may not all be made simultaneously.

The objective functions used in previous FBA studies (such as in [21]) have been capturing the optimal production of the biomass, which implicitly fixes the proportions of the different components of the biomass. The objective function **c**_2_ captures an analogous scenario, where the total mycolate content can be considered as the biomass. We feel that **c**_1_ reflects the biological situation more closely than **c**_2_ for this analysis for the following reasons: (a) it has been reported [4] that the mycobacterial cell can survive in the absence of one of the mycolate components, which cannot be accounted by **c**_2_; (b) the objective function **c**_2_ directly precludes the possibility of the cell surviving in the absence of even a single mycolic acid; for any genotype, **c**_2_ necessitates that the mycolates be produced in an all-or-none fashion in the corresponding phenotype. On the other hand, **c**_1_ favours the production of the most important mycolate that can be produced under the given conditions (i.e., following some gene deletions); and (c) reported biological data [44] suggest that the three major mycolates are in fact made at different phases of cell growth.

However, to make the analysis comprehensive, both objective functions have been used independently and the results presented. In the first case, the objective function **c**_1_ (Equation 2) accounts for the relative importance of the mycolates. The coefficients are indicative of the precise ranking order of the various mycolates, based on cell wall composition. On the other hand, the objective function in the second case, **c**_2_ (Equation 3), fixes the relative ratios of the mycolates based on the absolute values of the coefficients.

**Figure 3:**
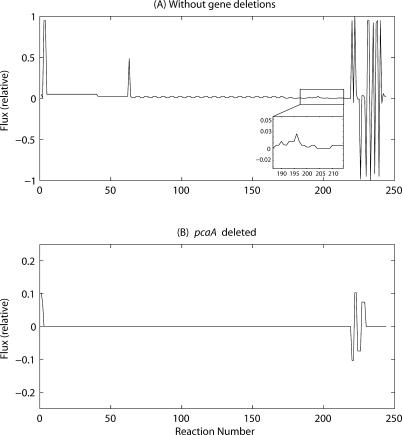
Flux Distributions Obtained from FBA Using the MAP Model and Objective Function **c**_2_ (A) in an unperturbed state, (B) upon deletion of *pcaA*. Inset in A refers to an enlarged version of the indicated portion. Note that the scale for (B) is different. It may be noted that the lines joining the various flux points have been drawn to aid in discerning the flux peaks clearly; the lines as such have no physical significance.

Once an objective function is fixed, the system translates to solving a linear programming (LP) problem as in Equation 7 (listed in Materials and Methods). The solution of the LP problem, using objective functions **c**_1_ and **c**_2_ yields flux distributions specifying the fluxes of all the internal reactions and the exchanges, as shown in Figures 2A and 3A, respectively. Both figures indicate two major peaks at reactions 3–4 and 63, apart from intense peaks at reactions 220–247. The peaks at reactions 3–4 and 63 correspond to the production of metabolites BCCP-biotin, malonyl CoA, and malonyl acyl carrier protein. The positive and negative peaks at reactions 220–247 correspond to the exchange fluxes originating from external metabolites such as ATP, NADP, NADPH, and CO_2_, indicating their exit from or entry into the MAP system, respectively. A repetitive pattern is observed for reactions 65–172, which is comprehensible in view of the cyclic nature of the reactions involved in extension of the carbon chain in FAS-II. Malonyl CoA is an important metabolite, since it is required not only for the formation of C_4_-acyl-ACP, but also for each of the ten steps of chain elongation by the FAS-I system, where the chain length of the fatty acid component of mycolic acid grows from four carbons to 24 carbons (see Figure 1). Besides, it is also required for the synthesis of malonyl acyl carrier protein, which in turn is required for chain elongation in each of the 20 chain elongation steps catalysed by the FAS-II system, where the chain length grows from C_16_ to C_52_–C_58_. The large fluxes seen for reaction 4, which produces malonyl CoA, and reaction 3, which produces BCCP-biotin, an immediate precursor of malonyl CoA, are explained by their high requirement. The exchange fluxes for external metabolites such as ATP, ADP, NADP, and NADPH are also understandably very high, since they are either utilised or produced in large quantities in the pathway.

While the flux distributions using either objective function are largely similar for the reactions belonging to sub-pathways A, B, and C (see Figure 1), significant differences are observed for the fluxes of the reactions producing mycolates and those immediately related to them. With objective function **c**_1_ (Figure 2A), a small peak at reaction 197 corresponding to α-mycolate is seen, as shown in more detail in the enlarged inset. It should be noted that, in this unperturbed state, the amounts of methoxy- and keto-mycolates produced are negligible, compared with that of α-mycolate. On the other hand, with objective function **c**_2_, all the mycolates are produced in the ratio mentioned earlier (Figure 3A, enlarged image in inset), as imposed by the objective function.

### Perturbations

#### Effects of in silico gene deletions, using **c**_1_.

The perturbations carried out on the MAP model using FBA were (a) in silico gene deletions and (b) inhibition by known drugs. Each of the 28 genes and hence its gene product was systematically deleted from the MAP model, one at a time, and its effect on the flux distribution was analysed (Table 2). Figures 2B and 2C are examples of flux distributions upon deletion of *inhA* and *pcaA*, respectively. Upon deletion of *inhA,* which catalyses every sixth reaction from 69 to 189 (see Figure 1C), the fluxes of almost all reactions were seen to be zero, except for reactions 1–2 and their corresponding external metabolites. On the other hand, deletion of *pcaA,* which is involved only in the production of α-mycolate (Figure 1D1), the flux pattern remained largely unaltered, except for an increase in the flux corresponding to *cis*-methoxy-mycolate (Figure 1D2). A flat flux distribution profile (of near zero) was observed upon deletion of 16 of the genes (and hence their gene products) in the MAP model. In D1, D2, and D3 sub-pathways, genes common to all three, such as *desA1,* fall into this category. On the other hand, genes responsible for the production of one or the other mycolate, such as *pcaA,* involved in D1, when deleted, did not significantly alter the overall flux distribution and are hence classified as non-essential. This is comprehensible, since in these cases, the system is still capable of producing the other two mycolates. Although it has been reported that all three mycolates in appropriate proportions are required for mycobacterial persistence and virulence, growth and survival to varying extents have been observed in the presence of any one component [4,45]. Experimental data available for *pcaA* deletion indeed show that the mutant bacilli can grow and survive for limited periods by producing a significant excess of keto-mycolate, to compensate for the absence of α-mycolate. Our results too show a similar compensatory effect upon deletion of *pcaA,* consistent with the experimental results about its non-essentiality [4,46]. In our results, however, methoxy- rather than keto-mycolate was produced in excess, because of the incorporation of their relative abundances available in literature [41] into the objective function. If the proportion of keto-mycolate was higher than that of methoxy-mycolate, then we would have observed a higher proportion of keto- rather than methoxy-mycolate, in our results. However, given the lack of more experimental details on the composition under different conditions, or the exact functional role of each of the mycolates, this difference does not seem too significant, at this stage.

**Table 2:**
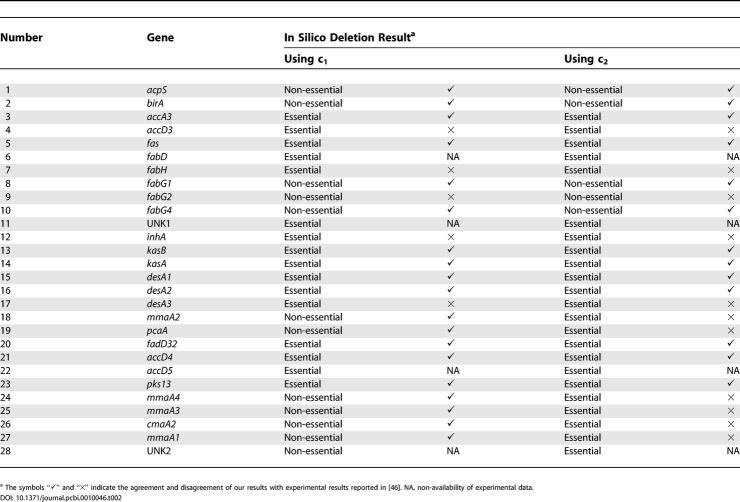
Results of In Silico Gene Deletion Studies for the 28 Genes in the MAP Model, Using Objective Functions **c**_1_ and **c**_2_

#### Effects of in silico gene deletions, using **c**_2_.

Systematic in silico gene deletion studies were carried out using the MAP model and with **c**_2_ as the objective function. Here too, each of the 28 genes and hence its product was systematically deleted from the MAP model, one at a time, and its effect on the flux distribution was analysed, as presented in Table 2. An example where a significant difference was found with respect to the perturbations using **c**_1_ is illustrated by *pcaA* deletion. Upon deletion of *pcaA,* using **c**_2_, almost all of the reaction fluxes were seen to have dropped to zero, except for reactions 1–2 and their corresponding external metabolites (Figure 3B). This is in contrast to the corresponding gene deletion, using **c**_1_, observed in Figure 2C, where the flux distribution is very similar to that in the unperturbed case. These results suggest *pcaA* to be essential if **c**_2_ is used, in contrast to the results obtained using **c**_1_ as well as reported biological data discussed above, indicating **c**_1_ to be a better objective function. Since the objective function **c**_2_ demands the production of all mycolates in the appropriate ratio, apart from *pcaA,* five other genes that were non-essential in the analysis using **c**_1_ were classified as essential.

Minimisation of metabolic adjustment (MOMA), using the methodology described by Segrè and co-workers [47], was also carried out for all the gene deletions (using **c**_1_ and **c**_2_ as objective functions in separate studies). There were no significant changes in the flux profiles, and hence in the interpretations, as compared with those described above.

Experimental data from systematic gene deletion studies using the transposon site hybridisation mutagenesis technique are available in the literature [46]. Comparison of such data for these 28 genes with the results obtained from our FBA study (using both objective functions) is shown in Table 2. With **c**_1_, a good correlation was observed for 19 genes, no experimental data was available for four genes, and disagreement was seen for only five genes. High correlation with experimentally observed data about the essentiality of individual genes indicates the usefulness of our MAP model and its study using FBA. With **c**_2_, a good correlation was observed for 13 genes and disagreement was seen for 11 genes.

On analysing the results of the gene deletion studies obtained using **c**_1_ and **c**_2_, it is apparent that the objective function **c**_1_ is able to reflect the biological situation better. **c**_2_ requires the production of all mycolates in a definite ratio under all conditions, which is not very appropriate, considering the fact that cells can survive even in the absence of one or more mycolates [4] and that in any state, only a single mycolate is produced in the cell. Hence, for the rest of the analyses, we have restricted the discussion to the gene deletion results obtained using **c**_1_.

The possible reasons for the disagreements (using **c**_1_) for *accD3, fabG2, fabH, inhA,* and *desA3* are discussed below. We had identified malonyl CoA as an internal metabolite, in the absence of concrete experimental evidence of its being produced by other reactions in the cell. However, if indeed malonyl CoA is produced by the cell through some other means, then AccD3 would no longer be essential, agreeing with the experimental results. *fabG2* has been reported as essential, while our analysis identified it as non-essential. Clearly, in our model, FabG2 can be substituted for by either FabG1 or FabG4. However, it may be possible that FabG2 could be responsible for catalysing some other critical reaction outside the MAP, which could contribute to its essentiality. *fabH* is a critical gene, whose product catalyses the important step that links FAS-I to FAS-II. Our sequence analysis studies also show that FabH has no homologues in the mycobacterial proteome. It is unclear as to why this is a non-essential gene in experimental studies. Similarly, *inhA,* identified as essential in our analysis but reported as non-essential in the experimental studies using transposon site hybridisation mutagenesis, is a well-known target for drugs such as isoniazid and ethionamide. While that may be the case for the conditions under which the transposon site hybridisation mutagenesis experiment was carried out, it is well-known that inhibition of InhA leads to a significant reduction in the growth of mycobacteria, making its inhibitors as front-line drugs. InhA is also known to be essential for mycolic acid synthesis [48], which in turn is known to be essential for survival of the pathogen [7]. In fact, InhA has been shown to be one of the few highly over-expressed proteins inside an infected macrophage [48]. The topology of the curated reaction network clearly makes InhA an essential gene for the system, which is in agreement with its known role for mycobacterial survival [48]. Another possibility could be that structural but not sequence homologues of InhA and FabH, which are not yet well-characterised (and hence not a part of our model), may substitute in their absence. *desA3* has been reported as non-essential but was identified as essential from our analysis. It is possible that our model may not have accounted for its exact physiological role, due to lack of information in literature.

Inhibition studies were carried out for isoniazid, since its inhibition of InhA has been well-characterised in the literature [9,10]. Inhibition in the context of FBA is in fact similar to that of deletion studies of the corresponding gene, except that the latter will lead to total inactivation of that gene, whereas inhibition by a drug need not necessarily lead to total inactivation. Just to represent the relative effect upon partial inactivation, we have considered a scenario where isoniazid would inhibit InhA to an extent of 90%. The flux profile shown in Figure 2D indicates much lower fluxes for each of the reactions. Similar results will be obtained for any inhibitor of InhA. Thus, the model and the method, besides being consistent with a requirement of a high percentage of inhibition for an ideal drug, also show their utility in analysing drug action when any quantitative data become available.

This method also has the potential to consider inhibition at multiple points. For example, isoniazid is thought to act at two points in the pathway (InhA and KasA), although conclusive experimental proof is still awaited [49]. The FBA study here presents a ready framework to analyse the effects of such drug inhibitions, which would be extremely difficult to judge by an inspection of the reaction map alone.

While the usefulness of FBA for large systems with high network connectivities and redundancy is well established, its application for specific pathways, which can be considered as simpler systems, has not yet been well explored in the literature. The study reported here illustrates the usefulness of FBA even for individual pathways. The effects of the perturbations to a system even of this size, either at single points or at multiple points, are beyond unambiguous comprehension and thus benefit from systematic studies such as FBA to get meaningful results. Moreover, FBA provides a handle to systematically identify essential genes in the pathway, irrespective of the size of the system, in a systematic, efficient, and much simplified manner. An advantage of considering specific pathways individually is that the objective functions for optimisation can be better defined, with specific biological relevance, to generate hypotheses useful for designing molecular biology studies quite readily. It must be noted that at the present time our understanding of systems, and hence their reconstructions in general, is not sufficient to generate knowledge that can replace biochemical or genetic experiments. However, an in silico framework for predicting gene essentiality, while complementing experimental data where available, has the additional advantage of enabling studies under various environmental conditions such as during low nutrition or upon oxidative stress, or even in the presence of drugs, which are difficult to perform experimentally.

### Identification of Drug Targets

Those genes that were classified as essential in the above analysis automatically form a first list of putative targets for anti-tubercular drugs, since their total inactivation results in loss of production of mycolic acids and hence the viability or the pathogenicity of the bacillus. However, it was reasoned that an ideal target should be essential not only in terms of the reaction it can catalyse, but also as the only protein coded by the genome that can perform the same task. Moreover, an ideal target should also have no recognisable homologue in the host system, which can in principle compete with the same drug, leading to unintended/adverse effects in the host system. Sequence analysis with the *M. tuberculosis* H37Rv and human proteomes was therefore carried out for each of the identified targets and the results are summarised in Table 3.

**Table 3:**
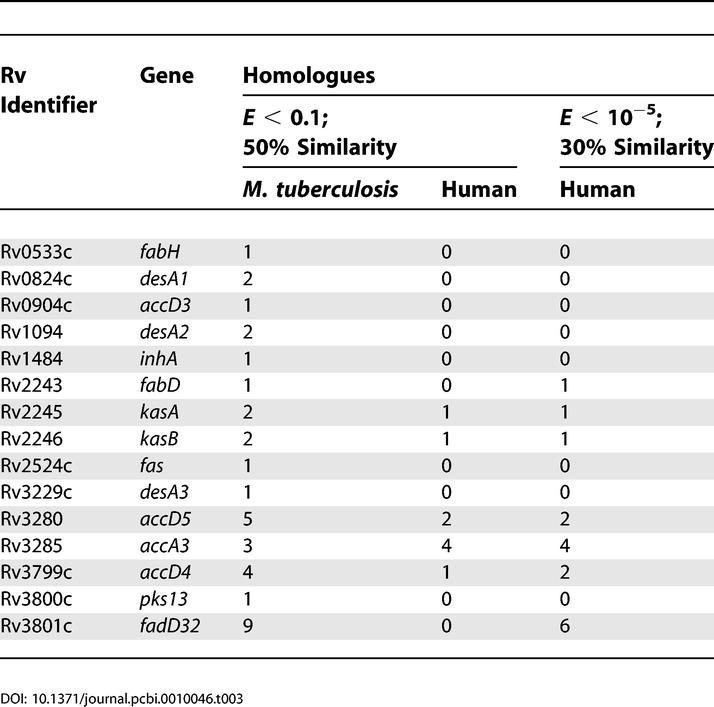
Homologues Present in *M. tuberculosis* and Human Proteomes for Genes Identified As Essential, Based on In Silico Gene Deletion Studies

Of the 16 proteins classified as essential in Table 2, no close homologues were observed within the *M. tuberculosis* H37Rv proteome for seven proteins: FabH, AccD3, InhA, FabD, Fas, Pks13, and DesA3. Similarities greater than 50% using the BLOSUM62 substitution matrix with an e-value of less than 0.1 for a length greater than 70% of the query sequence were considered as close homologues (in both proteomes). For identifying more distant/fast-evolving homologues in the human proteome, homologues were identified with a second set of criteria, considering similarities greater than 30% using the BLOSUM62 substitution matrix with an e-value of less than 10^−5^ for greater than 70% of the query sequence length. None of the seven proteins have close homologues in the human proteome. A distant homologue was identified with the second set of criteria for FabD. Those proteins, which have no other homologues either in the mycobacterial proteome or in the human proteome, are therefore obvious potential drug targets. Proteins lacking homologues with multiple cut-offs can be considered targets with greater confidence. Identification of their presence only in the bacterial cell helps in the process of validation as useful drug targets. Front-line anti-tubercular drugs in current clinical practice, isoniazid and ethionamide, in fact turn out to be inhibitors of InhA [9,10], preventing mycolic acid synthesis. Thus, the inhibition of the identified targets, which would all lead to impairment of mycolic acid synthesis, appears to be a promising strategy for designing anti-tubercular agents.

Besides the seven targets mentioned above, DesA1, DesA2, and FadD32 do not have any close homologues in the human proteome but have homologues in the *M. tuberculosis* H37Rv proteome. Of these, DesA1 and DesA2 are homologues of each other, but it is not clear as yet whether they are required together or if they can substitute for one another. KasA and KasB too are homologues of each other and do not share similarities with any other mycobacterial protein. However, they share considerable sequence similarities with a hypothetical human protein FLJ20604. FadD32 too exhibits distant homology with six other proteins in the human proteome. Such mycobacterial proteins would also be interesting drug targets, provided such homologies are considered during drug design.

In conclusion, the work presented here provides a framework to rationally identify targets for use in tuberculosis drug design and provides a ready shortlist that can be experimentally tested. This also outlines a general strategy for analysis of microbial metabolism, providing insights into targets for drug design. Systems approaches are being increasingly applied for understanding the metabolic capabilities of organisms, which can be exploited for drug design. A major bottleneck in this process is the accuracy of the model, which requires expert curation of available literature. The MAP model presented here should be of value not only in drug design but also for understanding mycolic acid synthesis in general. The model can also be adapted to perform quantitative simulations when kinetic data become available, and it can be used as a framework for incorporating newer or alternate components when such information becomes available.

## Materials and Methods

### Model building.

Initially, the Kyoto Encyclopedia of Genes and Genomes database was explored to obtain information about various proteins that comprise the MAP in *M. tuberculosis* H37Rv. Information about the FAS-I and FAS-II pathways in *M. tuberculosis* was available in the Kyoto Encyclopedia of Genes and Genomes database [50], but appeared to be based on pre-genomic annotations and was also incomplete and inconsistent in parts. The BioCyc repository of pathway models [51], on the other hand, had more recent annotations and provided a basic framework to build the MAP model. It contained information about 11 proteins. However, no explicit data were available about the specific reactions during the fatty acid elongation steps, catalysed by the FAS-I and FAS-II enzyme systems. Further, no information was available for the conversion of the FAS-II products to mycolates. Available literature (detailed in Table 1), as well as annotations in the TubercuList (http://genolist.pasteur.fr/TubercuList/) database, were therefore carefully analysed to fill the missing links and obtain a comprehensive model of the MAP, which contained information about 28 proteins, catalysing 219 reactions involving 197 metabolites, thus yielding a detailed pathway landscape. The model was encoded using SBML Level 2 Version 1 [52].

The set of reactions in the landscape were then mathematically represented as a stoichiometric matrix, **S** *_m × n_*, with every metabolite being represented by a row (*m* metabolites) and every reaction by a column (*n* reactions). The entries in each column correspond to the stoichiometric coefficients of the metabolites (negative for reactants and positive for products) for each reaction. The *i^th^* row of the matrix defines the participation of a particular metabolite across all metabolic reactions, and the *j^th^* column provides the stoichiometry of all metabolites in that reaction. Exchange fluxes were considered for metabolites that are typically exchanged with the environment (e.g., ATP, ADP, NAD[P], and NAD[P]H) and those that are not produced by the system, or those that are produced in the system for use in other metabolic pathways. Such metabolites, referred to as external metabolites, will have corresponding exchange fluxes in the pathway. The other metabolites were regarded as internal, which are produced by the system and consumed within the system itself. The external metabolites in the MAP model were identified manually. A list of external metabolites has also been supplied as part of Dataset S2.

### FBA.

FBA [12,53,54] has been shown to be a very useful technique for analysis of metabolic capabilities of cellular systems [20,21,55,56]. FBA involves carrying out a steady-state analysis, using the stoichiometric matrix for the system in question. The system is assumed to be optimised with respect to functions such as maximisation of biomass production or minimisation of nutrient utilisation, following which it is solved to obtain a steady-state flux distribution. This flux distribution is then used to interpret the metabolic capabilities of the system. The dynamic mass balance of the metabolic system is described using the stoichiometric matrix, relating the flux rates of enzymatic reactions, **v** *_n_*_ × 1_ to time derivatives of metabolite concentrations, **x** *_m_*_ × 1_ as


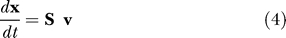



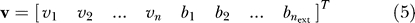


where *v_i_* signifies the internal fluxes, *b_i_* represents the exchange fluxes in the system, and *n*_ext_ is the number of external metabolites in the system. At steady-state,


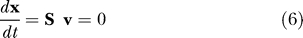


Therefore, the required flux distribution belongs to the null space of **S**. Since *m* < *n*, the system is under-determined and may be solved for **v** fixing an optimisation criterion, following which, the system translates into an LP problem:


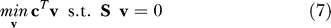


where **c** represents the objective function composition, in terms of the fluxes. Further, we can constrain:


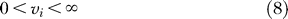



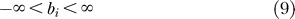


which necessitates all internal irreversible reactions to have a flux in the positive direction and allows exchange fluxes to be in either direction. Practically, a finite upper bound can be imposed, so that the problem does not become unbounded. This upper bound may also be decided based on the knowledge of cellular physiology. In our analysis, the upper bound was set as unity, which then effectively gives the relative ratios of the reaction fluxes. MATLAB (http://www.mathworks.com/) was used for solving the LP problem. The “linprog” routine from the Optimization Toolbox was used, which uses a large-scale interior point algorithm.

### Perturbations.

FBA also has the capabilities to address the effect of gene deletions and other types of perturbations on the system. Gene deletion studies were performed by constraining the reaction flux(es) corresponding to the gene(s) (and therefore, of their corresponding proteins[s]), to zero. Effects of inhibitors of particular proteins were also studied in a similar way, by constraining the upper bounds of their fluxes to any defined fraction of the normal flux, corresponding to the extents of inhibition.

### MOMA.

MOMA [18,47] is a technique similar to FBA, particular to the analysis of perturbed systems and has been reported to outperform FBA in certain cases. MOMA circumvents the use of an objective function for optimisation under perturbed conditions and rather solves for a flux distribution closest to the unperturbed system, subject to the new constraints imposed, minimising the metabolic adjustment of the system.

Analysis of the effects of deletion of individual genes on the flux profiles of the five mycolates provided us a handle to define essential and non-essential genes. Those deletions that resulted in zero or near-zero fluxes of all the mycolates were considered as essential, and the rest were considered as non-essential.

### Feasibility analysis of putative targets.

Sequence analysis was carried out using BLAST [57] to adjudge the feasibility of the putative targets identified through FBA. Homologues were searched for within the *M. tuberculosis* H37Rv and human proteomes, using BLOSUM62 substitution matrix. The BLAST outputs were parsed with home-grown scripts using BioPython modules, to identify homologues that satisfied various length and similarity criteria.

## Supporting Information

Dataset S1 SBML Model of the MAP An SBML model for the MAP in *M. tuberculosis*.
(124 KB XML)

Dataset S2 Flat File Containing Reactions in the MAP A flat file containing all the reactions in the MAP, along with the genes corresponding to proteins that catalyse the reactions. The external metabolites have also been indicated in the file.
(19 KB TXT)

### Accession Number

The SwissPROT/TrEMBL (http://www.ebi.ac.uk/trembl/) accession number for hypothetical human protein FLJ20604 is Q9NWU1. Gene accession numbers are listed in Table 1.

## Abbreviations

FAS-I: fatty acid synthase-I
FAS-II: fatty acid synthase-II
FBA: flux balance analysis
LP: linear programming
MAP: mycolic acid pathway
MOMA: minimisation of metabolic adjustment
SBML: Systems Biology Markup Language

## References

[pcbi-0010046-b01] Smith I (2003). *Mycobacterium tuberculosis*: Pathogenesis and molecular determinants of virulence. Clin Microbiol Rev.

[pcbi-0010046-b02] Barry CE III, Lee RE, Mdluli K, Simpson AE, Schroeder BG, et al. (1998). Mycolic acids: Structure, biosynthesis and physiological functions. Prog Lipid Res.

[pcbi-0010046-b03] Dubnau E, Chan J, Raynaud C, Mohan V, Lanéelle M, et al. (2000). Oxygenated mycolic acids are necessary for virulence of *Mycobacterium tuberculosis* in mice. Mol Microbiol.

[pcbi-0010046-b04] Glickman M, Cox J, Jacobs W (2000). A novel mycolic acid cyclopropane synthetase is required for cording, persistence, and virulence of *Mycobacterium tuberculosis*. Mol Cell.

[pcbi-0010046-b05] Crick DC, Mahapatra S, Brennan PJ (2001). Biosynthesis of the arabinogalactan-peptidoglycan complex of *Mycobacterium tuberculosis*. Glycobiology.

[pcbi-0010046-b06] Takayama K, Wang C, Besra GS (2005). Pathway to synthesis and processing of mycolic acids in *Mycobacterium tuberculosis*. Clin Microbiol Rev.

[pcbi-0010046-b07] Draper P, Daffé M, Cole ST (2005). The cell envelope of *Mycobacterium tuberculosis* with special reference to the capsule and outer permeability barrier. Tuberculosis and the tubercle bacillus.

[pcbi-0010046-b08] Pasqualoto KFM, Ferreira EI, Santos-Filho OA, Hopfinger AJ (2004). Rational design of new antituberculosis agents: Receptor-independent four-dimensional quantitative structure-activity relationship analysis of a set of isoniazid derivatives. J Med Chem.

[pcbi-0010046-b09] Lei B, Wei C, Tu S (2000). Action mechanism of antitubercular isoniazid. Activation by *Mycobacterium tuberculosis* KatG, isolation, and characterization of *inhA* inhibitor. J Biol Chem.

[pcbi-0010046-b10] Banerjee A, Dubnau E, Quemard A, Balasubramanian V, Um K, et al. (1994). *inhA*, a gene encoding a target for isoniazid and ethionamide in *Mycobacterium tuberculosis*. Science.

[pcbi-0010046-b11] Ma H, Zeng AP (2003). Reconstruction of metabolic networks from genome data and analysis of their global structure for various organisms. Bioinformatics.

[pcbi-0010046-b12] Bonarius HPJ, Schmid G, Tramper J (1997). Flux analysis of underdetermined metabolic networks: The quest for the missing constraints. Trends Biotech.

[pcbi-0010046-b13] Schuster S, Dandekar T, Fell DA (1999). Detection of elementary flux modes in biochemical networks: A promising tool for pathway analysis and metabolic engineering. Trends Biotech.

[pcbi-0010046-b14] Voit EO (2000). Computational analysis of biochemical systems: A practical guide for biochemists and molecular biologists.

[pcbi-0010046-b15] Stelling J, Klamt S, Bettenbrock K, Schuster S, Gilles ED (2002). Metabolic network structure determines key aspects of functionality and regulation. Nature.

[pcbi-0010046-b16] Cornish-Bowden A, Hofmeyr JHS (2002). The role of stoichiometric analysis in studies of metabolism: An example. J Theor Biol.

[pcbi-0010046-b17] Price ND, Papin JA, Schilling CH, Palsson BO (2003). Genome-scale microbial *in silico* models: The constraints-based approach. Trends Biotech.

[pcbi-0010046-b18] Segrè D, Zucker J, Katz J, Lin X, D'haeseleer P, et al. (2003). From annotated genomes to metabolic flux models and kinetic parameter fitting. OMICS.

[pcbi-0010046-b19] Edwards JS, Palsson BO (1999). Systems properties of the *Haemophilus influenzae* Rd metabolic genotype. J Biol Chem.

[pcbi-0010046-b20] Forster J, Famili I, Fu P, Palsson BO, Nielsen J (2003). Genome-scale reconstruction of the *Saccharomyces cerevisiae* metabolic network. Genome Res.

[pcbi-0010046-b21] Edwards JS, Palsson BO (2000). The *Escherichia coli* MG1655 *in silico* metabolic genotype: Its definition, characteristics, and capabilities. Proc Natl Acad Sci U S A.

[pcbi-0010046-b22] Becker SA, Palsson BO (2005). Genome-scale reconstruction of the metabolic network in *Staphylococcus aureus* N315: An initial draft to the two-dimensional annotation. BMC Microbiology.

[pcbi-0010046-b23] Schweizer E, Hofmann J (2004). Microbial type I fatty acid synthases (FAS): Major players in a network of cellular FAS systems. Microbiol Mol Biol Rev.

[pcbi-0010046-b24] Kikuchi S, Rainwater DL, Kolattukudy P (1992). Purification and characterization of an unusually large fatty acid synthase from *Mycobacterium tuberculosis* var. *bovis BCG*. Arch Biochem and Biophys.

[pcbi-0010046-b25] Choi KH, Kremer L, Besra GS, Rock CO (2000). Identification and substrate specificity of β-ketoacyl (acyl carrier protein) synthase III (mtFabH) from *Mycobacterium tuberculosis*. J Biol Chem.

[pcbi-0010046-b26] Scarsdale JN, Kazanina G, He X, Reynolds KA, Wright HT (2001). Crystal structure of the *Mycobacterium tuberculosis* β-ketoacyl-acyl carrier protein synthase III. J Biol Chem.

[pcbi-0010046-b27] Cole ST, Brosch R, Parkhill J, Garnier T, Churcher C, et al. (1998). Deciphering the biology of *Mycobacterium tuberculosis* from the complete genome sequence. Nature.

[pcbi-0010046-b28] Kremer L, Dover L, Carrère S, Nampoothiri K, Lesjean S, et al. (2002). Mycolic acid biosynthesis and enzymic characterization of the beta-ketoacyl-ACP synthase A-condensing enzyme from *Mycobacterium tuberculosis*. Biochem J.

[pcbi-0010046-b29] Marrakchi H, Ducasse S, Labesse G, Montrozier H, Margeat E, et al. (2002). MabA (FabG1), a *Mycobacterium tuberculosis* protein involved in the long-chain fatty acid elongation system FAS-II. Microbiology.

[pcbi-0010046-b30] Quémard A, Sacchettini JC, Dessen A, Vilcheze C, Bittman R, et al. (1995). Enzymatic characterization of the target for isoniazid in *Mycobacterium tuberculosis*. Biochemistry.

[pcbi-0010046-b31] Schaeffer ML, Agnihotri G, Volker C, Kallender H, Brennan PJ, et al. (2001). Purification and biochemical characterization of the *Mycobacterium tuberculosis* β-ketoacyl-acyl carrier protein synthases KasA and KasB. J Biol Chem.

[pcbi-0010046-b32] Phetsuksiri B, Jackson M, Scherman H, McNeil M, Besra GS, et al. (2003). Unique mechanism of action of the thiourea drug isoxyl on *Mycobacterium tuberculosis*. J Biol Chem.

[pcbi-0010046-b33] Glickman M, Cahill S, Jacobs W (2001). The *Mycobacterium tuberculosis cmaA2* gene encodes a mycolic acid trans-cyclopropane synthetase. J Biol Chem.

[pcbi-0010046-b34] Dinadayala P, Laval F, Raynaud C, Lemassu A, Laneelle M (2003). Tracking the putative biosynthetic precursors of oxygenated mycolates of *Mycobacterium tuberculosis*. J Biol Chem.

[pcbi-0010046-b35] George K, Yuan Y, Sherman D, Barry C (1995). The biosynthesis of cyclopropanated mycolic acids in *Mycobacterium tuberculosis*. Identification and functional analysis of CMAS-2. J Biol Chem.

[pcbi-0010046-b36] Glickman MS (2003). The *mmaA2* gene of *Mycobacterium tuberculosis* encodes the distal cyclopropane synthase of the α-mycolic acid. J Biol Chem.

[pcbi-0010046-b37] Yuan Y, Barry CE (1996). A common mechanism for the biosynthesis of methoxy and cyclopropyl mycolic acids in *Mycobacterium tuberculosis*. Proc Natl Acad Sci U S A.

[pcbi-0010046-b38] Yuan Y, Lee R, Besra G, Belisle J, Barry C (1995). Identification of a gene involved in the biosynthesis of cyclopropanated mycolic acids in *Mycobacterium tuberculosis*. Proc Natl Acad Sci U S A.

[pcbi-0010046-b39] Yuan Y, Crane D, Musser J, Sreevatsan S, Barry C (1997). MMAS-1, the branch point between *cis*- and *trans*-cyclopropane-containing oxygenated mycolates in *Mycobacterium tuberculosis*. J Biol Chem.

[pcbi-0010046-b40] Portevin D, de Sousa-D'Auria C, Montrozier H, Houssin C, Stella A (2005). The acyl-AMP ligase FadD32 and AccD4-containing acyl-CoA carboxylase are required for the synthesis of mycolic acids and essential for mycobacterial growth. J Biol Chem.

[pcbi-0010046-b41] Watanabe M, Aoyagi Y, Ridell M, Minnikin D (2001). Separation and characterization of individual mycolic acids in representative mycobacteria. Microbiology.

[pcbi-0010046-b42] Takayama K, Wang L, David HL (1972). Effect of isoniazid on the *in vivo* mycolic acid synthesis, cell growth, and viability of *Mycobacterium tuberculosis*. Antimicrob Agents Chemother.

[pcbi-0010046-b43] Vilchèze C, Morbidoni HR, Weisbrod TR, Iwamoto H, Kuo M (2000). Inactivation of the *inhA*-encoded fatty acid synthase II (FASII) enoyl-acyl carrier protein reductase induces accumulation of the FASI end products and cell lysis of *Mycobacterium smegmatis*. J Bacteriol.

[pcbi-0010046-b44] Yuan Y, Zhu Y, Crane DD, Barry CE (1998). The effect of oxygenated mycolic acid composition on cell wall function and macrophage growth in *Mycobacterium tuberculosis*. Mol Microbiol.

[pcbi-0010046-b45] Watanabe M, Aoyagi Y, Mitome H, Fujita T, Naoki H (2002). Location of functional groups in mycobacterial meromycolate chains; the recognition of new structural principles in mycolic acids. Microbiology.

[pcbi-0010046-b46] Sassetti CM, Boyd DH, Rubin EJ (2003). Genes required for mycobacterial growth defined by high density mutagenesis. Mol Microbiol.

[pcbi-0010046-b47] Segrè D, Vitkup D, Church GM (2002). Analysis of optimality in natural and perturbed metabolic networks. Proc Natl Acad Sci U S A.

[pcbi-0010046-b48] Monahan IM, Betts J, Banerjee DK, Butcher PD (2001). Differential expression of mycobacterial proteins following phagocytosis by macrophages. Microbiology.

[pcbi-0010046-b49] Mdluli K, Slayden RA, Zhu Y, Ramaswamy S, Pan X (1998). Inhibition of a *Mycobacterium tuberculosis* β-ketoacyl ACP synthase by isoniazid. Science.

[pcbi-0010046-b50] Kanehisa M, Goto S, Kawashima S, Okuno Y, Hattori M (2003). The KEGG resource for deciphering the genome. Nucleic Acids Res.

[pcbi-0010046-b51] Karp PD, Paley S, Romero P (2002). The pathway tools software. Bioinformatics.

[pcbi-0010046-b52] Hucka M, Finney A, Bornstein B, Keating S, Shapiro B (2004). Evolving a lingua franca and associated software infrastructure for computational systems biology: The Systems Biology Markup Language (SBML) project. Syst Biol.

[pcbi-0010046-b53] Edwards JS, Covert M, Palsson BO (2002). Metabolic modelling of microbes: The flux-balance approach. Environ Microbiol.

[pcbi-0010046-b54] Kauffman KJ, Prakash P, Edwards JS (2003). Advances in flux balance analysis. Curr Opin Biotech.

[pcbi-0010046-b55] Alvarez-Vasquez F, Sims K, Cowart L, Okamoto Y, Voit E (2005). Simulation and validation of modelled sphingolipid metabolism in *Saccharomyces cerevisiae*. Nature.

[pcbi-0010046-b56] Edwards JS, Ibarra RU, Palsson BO (2001). *In silico* predictions of *Escherichia coli* metabolic capabilities are consistent with experimental data. Nat Biotechnol.

[pcbi-0010046-b57] Altschul SF, Madden TL, Schäffer AA, Zhang J, Zhang Z (1997). Gapped BLAST and PSI-BLAST: A new generation of protein database search programs. Nucleic Acids Res.

[pcbi-0010046-b58] Kremer L, Nampoothiri KM, Lesjean S, Dover LG, Grahami S (2001). Biochemical characterization of acyl carrier protein (AcpM) and malonyl-CoA:AcpM transacylase (mtFabD), two major components of *Mycobacterium tuberculosis* fatty acid synthase II. J Biol Chem.

[pcbi-0010046-b59] Portevin D, De Sousa-D'Auria C, Houssin C, Grimaldi C, Chami M (2004). A polyketide synthase catalyzes the last condensation step of mycolic acid biosynthesis in mycobacteria and related organisms. Proc Natl Acad Sci U S A.

